# Increased risk of type I errors for detecting heterogeneity of treatment effects in cluster-randomized trials using mixed-effect models

**DOI:** 10.1186/s12874-025-02744-6

**Published:** 2026-01-12

**Authors:** Noorie Hyun, Abisola E Idu, Andrea J. Cook, Jennifer F. Bobb

**Affiliations:** 1 Kaiser Permanente Washington Health Research Institute, 1730 Minor Ave., Ste 1360, Seattle, WA 98101 USA; 2 Department of Biostatistics, School of Public Health, University of Washington, Seattle, Washington, USA

**Keywords:** Heterogeneity of treatment effects, Mixed-effects model, Generalized estimating equation, Cluster randomized trials, Heterogeneous correlations across subgroups

## Abstract

**Background/Aims:**

Evaluating heterogeneity of treatment effects (HTE) across subgroups is common in both randomized trials and observational studies. Although several statistical challenges of HTE analyses including low statistical power and multiple comparisons are widely acknowledged, issues specific to clustered data, including cluster randomized trials (CRTs), have received less attention. For testing interactions in linear mixed-effects models (LMM), Barr et al. (2013) suggested that: random slopes for interaction terms should be studied. In this paper, we explore the impact of model misspecification, including generalized LMM (GLMM) with or without random slopes, and provide recommendations for conducting inference for HTE across subgroups in CRTs.

**Methods:**

We conducted a simulation study to evaluate the performance of common analytic approaches for testing the presence of HTE for continuous, binary, and count outcomes: generalized linear mixed models (GLMM) and generalized estimating equations (GEE) including interaction terms between treatment and subgroup. Several simulation scenarios covered broad range of scenarios in CRTs, for example, small to a large number of clusters, small to moderate cluster-specific random slopes for subgroup. The performance metric was the empirical type I error rate compared to a nominal level. We applied the analytical methods to a real-world CRT using the count outcome utilization of healthcare from the motivating Primary Care Opioid Use Disorder treatment (PROUD) trial.

**Results:**

We found that standard GLMM analyses that assume a common correlation of participants within clusters can lead to severely elevated type 1 error rates of up to 47.2% compared to the 5% nominal level if the within-cluster correlation varies across subgroups. A maximal GLMM, which allows subgroup-specific within-cluster correlations, achieved the nominal type 1 error rate, as did GEE (though rates were slightly elevated even with as many as 50 clusters). Applying the methods to the real-world CRT, we found a large impact of the model specification on inference.

**Conclusions:**

We recommend that HTE analyses using the maximal GLMM account for within-subgroup correlation to avoid anti-conservative inference. For Wald t-testing of HTE in small sample clusters, appropriate small sample correction methods should be considered based on the outcome data type.

**Supplementary Information:**

The online version contains supplementary material available at 10.1186/s12874-025-02744-6.

## Background

Dealing with clustered data is extremely common in public health research. It can arise by design including cluster randomized trials (CRTs) or due to the hierarchical structure of the data (e.g. multiple outcomes per participant, patients clustered within providers, or providers clustered within clinics) [1, 2]. Addressing correlation of clustered outcome data in the analysis is critical to correctly estimate standard errors and avoid inflated type I error.

Model-based methods for analyzing clustered data can be broadly classified into conditional and marginal models according to the interpretation of the model parameters [2, 3]. Conditional models adjust for cluster-specific effects in estimating the intervention effect, and the model parameters are usually estimated by mixed-effects regression, such as generalized linear mixed models (GLMM). In contrast, marginal models, such as generalized estimating equations (GEE), are often employed when focusing on population-level effects and interpreting the intervention effect coefficient as a population-averaged effect.

Beyond estimating overall treatment effects within the study population, many studies also examine differences in treatment effects across subgroups. These analyses are often referred to as effect modification or heterogeneity of treatment effect (HTE) analyses. For randomized controlled trials (RCTs), subgroup analyses may be conducted to satisfy regulatory requirements to evaluate whether efficacy is consistent across subgroups or to identify whether safety problems are constrained to specific subgroups [4–6]. Alternatively, subgroup analyses may be conducted for exploratory purposes to identify subgroups most likely to benefit from an intervention [7]. Regression models are often used for HTE analysis that include the subgroup factor, the intervention group, and their interaction term(s), as well as any additional pre-specified covariates [8].

**Fig. 1:**
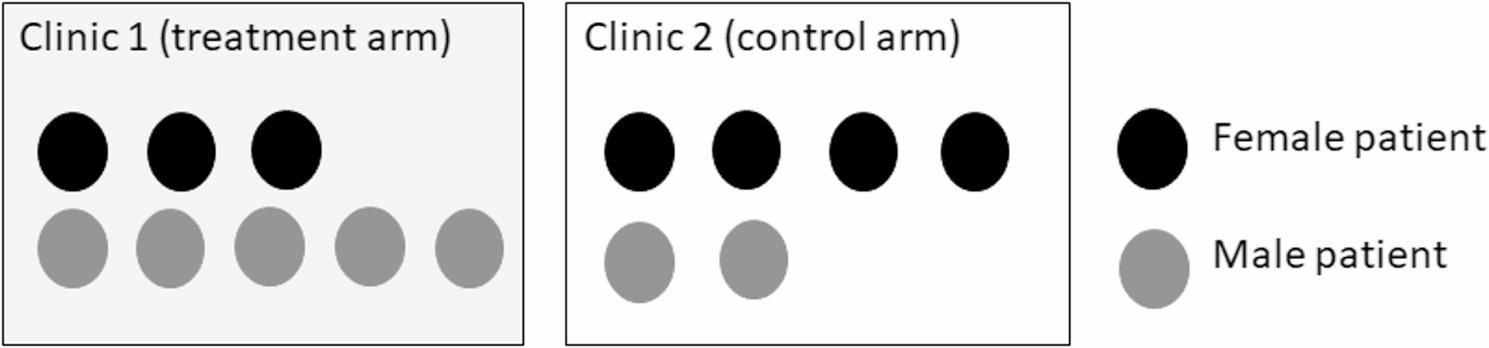
Illustration of an example of two arms with two clusters in a typical cluster-randomized trial, in which patients are classified within sex-subgroups (here females and males are represented by black and dark grey disks, respectively) and are nested within clinic (cluster) with cluster-level treatment group assignment. In addition to the clustering of patients within subgroups and clinics, the sample size varies across clinics, both overall and within subgroups

Most RCT literature on statistical approaches for HTE analyses addresses analytical issues observed in individually randomized trials without clustering. These issues include lack of power, multiple testing and the potential for false positive findings, and the limited interpretation of the marginal subgroup analyses from the standpoint of patients belonging to a combination of subgroup factors. In contrast, analytical issues regarding subgroup analyses for clustered data have received comparatively little attention. The correlation structure of subgroups nested within clusters (see Fig. 1) could lead to statistical challenges in detecting HTE across subgroups using model-based methods. The cluster-nested subgroup specific correlation can be parametrized by random slopes for subgroup dummy variables. For testing interactions in linear mixed-effects models, Barr et al. [9, 10] suggested that by-cluster random slopes for any interactions should be studied where all factors comprising the interactions are within-cluster. However, these prior papers were targeted to experimental psychology field rather than CRTs. Thus, this potential for elevated type I error rates in the HTE analyses in CRTs may be underappreciated. Moreover, non-continuous outcomes and small numbers of clusters have not been considered. 

In particular, type I error inflation was observed in HTE analyses using GLMM conducted as part of the Primary Care Opioid Use Disorder treatment (PROUD) trial, a pragmatic, two-arm, cluster-randomized trial in 12 clinics that tested whether implementing nurse care management for opioid use disorder (OUD)—a clinic-level intervention—increased OUD treatment in diverse primary care settings in the United States [11]. In pre-planned HTE analyses of the main effectiveness (count) outcome, which used a simple GLMM with only a random intercept for cluster, the study estimated similar, almost identical, within-subgroup intervention effects with large 95% confidence intervals, but extremely small yet statistically significant interaction effects between intervention and subgroups.

In this paper, we sought to examine the impact of the choice of modeling approach (GEE and GLMM with and without random slopes for subgroups) on statistical inference and type I error rate for HTE across subgroups in general CRT settings with continuous, count and binary outcome types. We consider the scenario in which the within-cluster correlation differs across the subgroups being examined, and we propose a maximal GLMM allowing cluster-nested subgroups’ correlation in Methods Section. To examine the impact of model (mis)specification, we conduct a simulation study in which we compare results of both GEE and GLMM models with different correlation structures and across different outcome types. Application to the motivating PROUD study is also presented.

## Methods

We consider the setting of a parallel-group CRT with two arms, $\:I$ clusters and cluster size $\:{n}_{i}$ for cluster $\:i.\:$Observed are outcome measurements $\:{Y}_{ij}$ from subject $\:j$ within cluster $\:i$, binary indicator $\:tr{t}_{i}$ for the assigned intervention or control to cluster $\:i$, and a vector of (p-1) dummy variables for a subgroup factor $\:{g}{{r}}_{{i}{j}}$. For simplicity, we assume a simple unadjusted model including only the subgroup of interest, but additional covariates could be straightforwardly incorporated as desired.

### GEE and GLMM

We first present two traditional models applied to CRTs. For GEE, the mean function is given by

1 $$\:g\left\{E\right({Y}_{ij}\left)\right\}\:={\beta\:}_{0}+{trt}_{i}{\beta\:}_{trt}+{g}{{r}}_{ij}^{{T}}{{\beta\:}}_{gr}+{\left(tr{t}_{i}\times\:{g}{{r}}_{ij}\right)}^{{T}}{{\beta\:}}_{{m}{o}{d}}\,\,\,\mathrm{.}$$

We consider canonical links for $\:g(\cdot\:)$, that is, the identity, log and logit functions for continuous, count and binary outcomes, respectively. The regression coefficient $\:{\beta\:}_{trt}$ is the intervention effect within the reference subgroup, and $\:{{\beta\:}}_{{m}{o}{d}}$ is the vector parametrizing the difference in intervention effects comparing the other subgroup categories to the reference subgroup (on the difference, log relative risk, or log odds ratio scale, depending on the link function). Any non-zero components of $\:{{\beta\:}}_{{m}{o}{d}}$ correspond to subgroups showing heterogenous intervention effects.

For an asymptotic variance estimator of GEE, we use the robust sandwich variance based on model-based information matrix and design-based empirical variance [12]. We used an independent working correlation in our simulation and application that is commonly used in practice to estimate an average intervention effect at the person-level [13]. Note that using other working correlation structures, such as exchangeable, may improve relative efficiency, but the advantage of GEE is the robustness to misspecified working correlation structures. We apply a z-test or F-test to assess HTE, depending on whether the number of clusters is moderate to large or small, respectively. By including GEE in our simulation, we sought to evaluate if using a simple correlation structure in practice achieved valid inferences in the HTE setting or if GEE would have similar issues as observed for GLMM. We use the ‘gee’ R-package version 4.13-29 to fit the GEE model.

For GLMM, the conditional mean function is similar to the GEE model above, but includes a cluster-specific random effect $\:{U}_{i}$:

2 $$\:g\left\{E\left({U}_{i}\right)\right\}\:={\beta\:}_{0}+{trt}_{i}{\beta\:}_{trt}+{g}{{r}}_{{i}{j}}^{{T}}{{\beta\:}}_{{g}{r}}+{\left(tr{t}_{i}\times\:{g}{{r}}_{{i}{j}}\right)}^{{T}}{{\beta\:}}_{{m}{o}{d}}+{U}_{i}\,\,\mathrm{,}$$

where $\:{U}_{i}\sim N\left(0,{\sigma\:}^{2}\right)$ are i.i.d. with finite variance. To estimate the model parameters, marginal log-likelihoods are calculated by integrating out the random effects; for non-identity link functions no closed form exists such that approximate methods are employed [14]. For computational efficiency in our simulation study, we used the Laplace approximation to calculate the marginal log-likelihoods (default setting in the ‘lmer4’ R-package used to fit mixed models for this study [15]). While both the likelihood ratio test (LRT) and Wald t-test can be considered for assessing HTE from GLMM, we primarily use the Wald t-test, except in cases where the LRT is necessary. This choice is motivated by the fact that the asymptotic chi-square distribution assumed for the LRT may poorly control the type I error rate, particularly when cluster sizes are large—as is the case in our motivating study.

### Maximal GLMM model

We propose a maximal GLMM that includes cluster-nested subgroup-specific correlations (called GLMM2). For this model, the conditional mean function is given by:

3 $$\:g\left\{E\left({{U}}_{i\:}\right)\right\}={\beta\:}_{0}+tr{t}_{i}{{\beta\:}}_{{t}{r}{t}}+{g}{{r}}_{{i}{j}}^{{T}}{{\beta\:}}_{{g}{r}}+{\left(tr{t}_{i}\times\:{g}{{r}}_{{i}{j}}\right)}^{{T}}{{\beta\:}}_{{m}{o}{d}}+{{g}{{r}}_{{i}{j}}^{{T}}{U}}_{{i}}\,\,\,\mathrm{,}$$

where each single component of random effects $\:{{U}}_{i} := \left({U}_{i1},{U}_{i2},\ldots\:,\:{U}_{ip}\right)\:$corresponds to the random effect due to subgroup $\:k$ in cluster $\:i$ for $\:k=1,\dots\:,\:p$, where $\:p$ is the number of subgroup levels. We assume that $\:{{U}}_{i}$ follows a multivariate normal distribution with zero mean and $\:p\:x\:p$ variance-covariance matrix $\:{\Sigma\:}$, which is positive-definite. We further assume that the $\:{{U}}_{i}$ are independent across clusters. It is also assumed that given $\:{{U}}_{{i}}$, $\:{Y}_{ij}$ is independent of $\:{Y}_{i{j}^{{\prime\:}}}\:$for any $\:j$ and $\:{j}^{{\prime\:}}$; $\:{Y}_{ij}$ is independent of $\:{Y}_{i{{\prime\:}j}^{{\prime\:}}}\:$for any $\:j$ and $\:{j}^{{\prime\:}}$ given different clusters $\:i$ and $\:{i}^{{\prime\:}}$. We consider the GLMM model [4] as a special case of the maximal GLMM [5] by restricting subgroup-specific random effects such that $\:{U}_{i1}={U}_{i2}=\dots\:=\:{U}_{ip}\:$. Under this restriction, the covariance matrix $\:{\Sigma\:}$ becomes $\:{\sigma\:}^{2}{J}_{p\times\:p}$, where $\:{J}_{p\times\:p}$ is $\:p\times\:p\:$matrix with all ones because $\:Cov\left({U}_{ik},\:{U}_{ik^{{\prime\:}}}\right)=Var\left({U}_{ik}\right)=Var\left({U}_{i{k}^{{\prime\:}}}\right)$ for any $\:1\le\:k,{k}^{{\prime\:}}\le\:p$. Then the subgroup-specific random intercepts in the maximal GLMM can be reparametrized by using a random intercept and random slope for the subgroup dummy variables. We consider canonical link functions and primarily apply Wald t-test as described in Sect. GEE and GLMM. We use the ‘lmer4’ R-package version 1.1–31 to fit the proposed model.

#### Singular model fitting issues and two-step procedure

Given the more complex correlation structure, the GLMM2 model may be more likely to encounter a singular fit than the standard GLMM introduced in Sect. GEE and GLMM. One approach when a singular fit occurs is to simply proceed with the fitted model estimates. An alternate approach is to apply a two-step procedure: after encountering a singular fit (e.g., due to one of the subgroup-specific random effects having variance estimated as 0), one proceeds by fitting the standard GLMM from Sect. GEE and GLMM that includes a common random intercept variance for all subgroups. We evaluate both approaches in the simulation study in the scenarios in which we observe singular fits occurring, including small random effects variances and/or small numbers of clusters. In practice, the true model is unknown, and one might consider testing for the presence of subgroup-specific random effects. However, such tests often lack sufficient power in CRTs, especially when the HTE analysis is neither confirmatory nor the primary objective. Therefore, we do not include a formal testing step in the two-step procedure.

Further, to avoid computational difficulties, we reparametrized the GLMM2 by using a random intercept and random slope parameterization for the subgroup dummy variables (see sample code in the Appendix). The reparametrized model reduces the occurrence of singularity issues defined by random effects estimates equal to 0. Although mathematically equivalent, we note that the two models that include subgroup specific random intercepts and random intercept and slope, respectively may not yield identical estimates due to the algorithm convergence. Although reparametrizing the GLMM2 results in less frequent singular fit issues, given the more complex correlation structure the GLMM2 is still more likely to yield a singular fit relative to the traditional GLMM model.

### Small cluster size

For settings with a small number of clusters (< 50), various methods for correcting inferences have been studied in the setting of no subgroup-specific correlations within cluster for GLMM and GEE [16, 9. However, small cluster correction methods for GLMM2 or for the setting of existing subgroup-specific correlations within cluster have not been systematically studied. Thus, we applied four existing correction methods to GLMM and GLMM2: (a) Satterthwaite degrees of freedom for the Wald t-test for continuous outcomes, (b) subtracting the number of cluster-level parameters for fixed effects from the number of clusters [17] for the Wald t-test statistics for binary and count outcomes, (c) “between-within” denominator degrees of freedom of freedom approximation for the Wald t-test statistics for binary and count outcomes [18], and (d) parametric bootstrapping 95% confidence interval based on 100 simulated datasets from the fitted model. The ‘parameters’ R-package was used to implement corrections (c) and (d). Given the computational burden of bootstrapping within a simulation study, we first applied (a), (b) and (c) to the GLMM2 and GLMM. If at least one of these methods yielded a type I error rate close to the nominal level ($\:4\%\sim5\%)$, we presented the results from the best performer from among these three methods. However, if none of the methods maintained the nominal level, we additionally applied the bootstrapping method and then chose the best performer (from all four methods). Lastly, we applied the Fay and Graubard correction in the GEE model by using a Wald-type F-test with the unadjusted sandwich variance and ‘ditilde’ degrees of freedom, as implemented by default in the ‘saws’ R package [19].

### Simulation study

We compared the proposed GLMM2 [3], with and without the two-step procedure when encountering singular fits, with GEE [1] and GLMM [4]. We primarily evaluated the methods in terms of the type I error rate for testing for effect modification between the intervention indicator and a subgroup factor (primarily focusing on a binary subgroup variable for simplicity). We set the nominal significance level to be 5%. In addition to the type I error rate, we also compared bias and empirical standard deviations. These latter comparisons focused on continuous and count outcomes as the three models’ regression coefficients for the effect modification have marginal interpretation, so those are comparable to each other. In contrast, in the logistic regression-due to the non-collapsibility of the odds ratios-the three models’ coefficients for effect modification estimate different quantities and are not comparable.

We considered seven different data generating scenarios (Scenarios 1–7) with parameter values shown in Table 1. These included three scenarios in which the GLMM2 was the true model: an “ideal” setting in which there are a large number of clusters and moderately sized random effects (Scenario 1), as well as more challenging settings with either small random effects (Scenario 2) or a small number of clusters (Scenario 3). We also evaluated scenarios in which the standard GLMM was the true data-generating model and GLMM2 was over fitted—namely, Scenarios 4, 5, and 7—across different regression parameters settings with small to moderate numbers of clusters. Finally, Scenario 6 was included to assess the robustness of GLMM2 under model misspecification: here, the true model followed a GLMM2 structure with heterogeneous covariances between both arms for subgroup-specific random effects, meaning that both GLMM and GLMM2 were misspecified. For each scenario, we conducted 1,000 simulation replicates. We applied each of the comparator approaches described in Sect. GEE and GLMM–Small Cluster Size to Scenarios 1–6. In Scenario 7, which examined power of methods, we compared only GLMM and GLMM2, as GEE is a marginal model and not directly comparable in terms of power.

In the simulation, we consider scenarios of CRTs with N clusters, 1:1 random assignment of clusters to two treatment groups (e.g., intervention or control), and a balanced subgroup factor within a cluster. For data generation, we define a binary subgroup indicator $\:g{r}_{ij}$, (0 vs. 1), which is independently generated from a Bernoulli distribution with 0.5 probability for Scenarios 1–4 and 6–7, and for Scenario 5, a three-level categorical subgroup with dummy variables ($\:g{r}_{ij1}$, $\:g{r}_{ij2})=$(0,0), (1,0), or (0,1), which is independently generated from a multinomial distribution with even probabilities 1/3. We independently generate $\:{U}_{i}\sim\mathrm{N}\left(0,{\sigma\:}^{2}\right)\:$if model [4] is the true model or $\:({U}_{i,1},{\dots\:,U}_{i,\mathrm{p}})$ if model [5] is the true model from a multivariate normal distribution with zero mean and $\:{\Sigma\:}$ covariance, $\:MVN(0,{\Sigma\:})$. In addition, for Scenario 6, we independently generate $\:({U}_{i,1},{U}_{i,2})$ from $\:MVN\left(0,{{\Sigma\:}}_{0}\right)$ if cluster $\:i$ is assigned to control group or $\:MVN\left(0,{{\Sigma\:}}_{1}\right)$ if cluster $\:i$ is assigned to intervention group, where $\:{{\Sigma\:}}_{0}\ne\:{{\Sigma\:}}_{1}$. When $\:{Y}_{ij}\:$is continuous, count and binary, we assume $\:{Y}_{ij}$ follows a normal distribution with an identity link, Poisson distribution with log link, and Bernoulli distribution with logit link, respectively.

Universal parameters used for most scenarios are described. Based on the estimated coefficients in our motivating PROUD dataset, for the null binary effect modification in Scenarios 1–4 and 6, we set the regression parameters $\:({\beta\:}_{0},{\beta\:}_{trt},{\beta\:}_{gr},{\beta\:}_{mod})$ to be $\:\left(\mathrm{0,0.5,0.3,0}\right)$ for the continuous outcome setting, $\:\left(-1,\:-0.07,\:-0.5,\:0\right)\:$for count outcomes, and (-1.66, -0.32, -0.08, 0) for binary outcomes. When considering dummy variables for a three-level categorical effect modifier, we set the regression parameters to be $\:({\beta\:}_{0},{\beta\:}_{trt},{\beta\:}_{gr1},{\beta\:}_{gr2},{\beta\:}_{mod,1},{\beta\:}_{mod,2})$=(0,0,0.5,0.3,0.15,0,0) for continuous outcomes, (-1,-0.07, -0.5,-0.3, 0,0) for count outcomes, and (-1.66, -0.32, -0.08, -0.05,0,0) for binary outcomes. For power comparison in Scenario 7, we set parameters $\:({{\upbeta\:}}_{0},{{\upbeta\:}}_{\mathrm{t}\mathrm{r}\mathrm{t}},{{\upbeta\:}}_{\mathrm{g}\mathrm{r}},{{\upbeta\:}}_{\mathrm{m}\mathrm{o}\mathrm{d}})$ to be $\:\left(\mathrm{0,0.5,0.3,0.2}\right)$ for the continuous outcomes, $\:\left(-1,\:-0.07,\:-0.5,\:0.1\right)\:$for count outcomes, and (-1.66, -0.32, -0.08, 0.15) for binary outcomes. The residual error variance for continuous outcomes was set to 0.64. In most scenarios, effect modification was assessed using a Wald t-test. Exceptionally, for Scenario 5, we conducted a global test of the null hypothesis such that $\:{H}_{0}:{\beta\:}_{mod,1}={\beta\:}_{mod,2}=0\:$using an LRT test as the LRT is more commonly used than the union of individual Wald tests when simultaneously testing multiple parameters. For settings with 12 clusters, we applied small-sample correction methods as outlined in Sect. Small Cluster Size. The specific correction methods used for scenarios with a small number of clusters (including Scenarios 3 and 4) are summarized in Table 2.

**Table 1:** Simulation setting by scenario

Scenario description	True model	Covariance$\:{\Sigma\:}$for$\:({\mathrm{U}}_{\mathrm{i},0},{\mathrm{U}}_{\mathrm{i},1})$or variance$\:{{\upsigma\:}}^{2}\:$for$\:{\mathrm{U}}_{\mathrm{i}}$	Number of clusters, *N*
Scenario 1: ideal settings for GLMM2 in terms of adequate random effect and number of clusters	GLMM2	$$c_{t}\left(\begin{array}{cc}0.2 & 0.13\,\\0.13 & 0.1\end{array}\right)$$, $$c_{t}\left(\begin{array}{cc}0.5 & 0.25\,\\0.25 & 0.5\end{array}\right)$$ and $$c_{t}\left(\begin{array}{cc}0.25 & 0.18\,\\0.18 & 0.5\end{array}\right)$$ for continuous, count and binary outcomes, respectively.	*N* = 50 and 100 with fixed cluster size 100.
Scenario 2: small random effects but adequate number of clusters	GLMM2	Covariances are downsized by 1/10 of the variances in Scenario 1 with the same correlation degrees. These covariances reflect PROUD data application.	*N* = 50 and 100 with fixed cluster size 100.
Scenario 3: adequate random effects but small number of clusters	GLMM2	The same covariances of Scenario 1	*N* = 12 with fixed cluster 100 and *N* = 12 with varying cluster size of (25, 50, 100, 150, 300).
Scenario 4: to evaluate the robustness of GLMM2 when GLMM2 is over fitted.	GLMM	$\upsigma\:^{2}$is 0.2, 0.5 and 0.5 for continuous, count and binary outcomes, respectively. i.e.,$$\:{\Sigma\:}={{\upsigma\:}}^{2}\left(\begin{array}{cc}1 & 1\,\\1 & 1\end{array}\right)$$.	*N* = 50 with fixed cluster 100 and *N* = 12 with varying cluster size of (25, 50, 100, 150, 300).
Scenario 5: to evaluate the robustness of GLMM2 when GLMM2 is over fitted, and subgroup is three-level categorical.	GLMM	The same covariances of Scenario 1.	The same setting of Scenario 4.
Scenario 6: to evaluate the robustness of GLMM2 when GLMM2 is misspecified.	GLMM2 with heterogeneous covariances between both arms for subgroup-specific random effects	$$c_{t}\left(\begin{array}{cc}0.2 & 0.13\,\\0.13 & 0.1\end{array}\right)$$ and $$c_{t}\left(\begin{array}{cc}0.5 & 0.25\,\\0.25 & 0.5\end{array}\right)$$ for continuous, count and binary outcomes, respectively, where *c*_*t*_ =1 for intervention arm and *c*_*t*_ =1.4 for control arm.	The same setting of Scenario 4.
Scenario 7: to compare power of GLMM and GLMM2 for detecting HTE.	GLMM	The same covariances of Scenario 1.	The same setting of Scenario 4.

**Table 2:** Selected small-sample correction methods for Wald t-test in simulation scenarios with a small number of clusters by modeling approach and outcome type

True model	Fitted model	Outcomes		
		Continuous	Count	Binary
GLMM2	GLMM2	Satterthwaite	Between-within	Subtracting the number of cluster-level parameters for fixed effects from the number of clusters
	GLMM		Subtracting the number of cluster-level parameters for fixed effects from the number of clusters	Subtracting the number of cluster-level parameters for fixed effects from the number of clusters
GLMM	GLMM2		Parametric bootstrap	Parametric bootstrap
	GLMM		Between-within	Between-within

Bias is calculated by the difference between the average of the point estimates and the true value; the empirical standard deviation is the standard deviation of the point estimates. Type I error rates are calculated as the proportion of rejections for effect modification at the nominal 0.05 level. For the parametric bootstrapping method, the percentage of 95% confidence intervals excluding 0, the null effect modification, was calculated.

### Real data illustration

We re-analyze an HTE analysis from the PROUD study which evaluated whether implementation of office-based addiction treatment (intervention)—relative to usual care—reduced acute care utilization among primary care patients with opioid use disorder (OUD). The PROUD trial was funded by the National Institute for Drug Abuse (NIDA) Clinical Trials Network (CTN) and was conducted in 12 clinics from six diverse health systems across the US from 2015 to 2020. Within each health system, one of two clinics was assigned to the intervention and the other to usual care. The patient-level effectiveness outcome is the number of days of utilization of acute care services, including emergency department visits, urgent care visits, and inpatient hospitalizations. Details of study design and primary outcome results have been previously published [11].

For this application, we focus on examining the potential for modification of the intervention effect by sex as documented in patients’ electronic health records. We employed GLMM2, GLMM, and GEE, using a Poisson distribution with log link to analyze the count outcomes. The GLMM accounted for the random intercept due to the 12 clinics, while the GLMM2 accounted for the random intercept due to the clinics and the random slope due to sex (female vs. male). In all models, we adjusted for the baseline value of the outcome (reflecting the study’s primary analysis approach). We applied the same correction techniques for small numbers of clusters (12 clinics) as described in Scenario 2, using the Wald t-test with between-within correction for GLMM2, with 10 degrees of freedom (= 12 − 2) for GLMM and GEE. Particularly, Fay and Graubard’s correction for the GEE model couldn’t be applied because of the singular information matrix. To align with the simulation study, we note that this reanalysis does not exactly replicate the primary outcome GLMM analysis, which used a likelihood ratio small-cluster test [20]. The 95% confidence intervals of the intervention effect within men and women were also small-cluster corrected by using the analogous methods used for p-value correction.

## Results

### Simulation results

The summary statistics of Scenarios 1–7 with 1,000 simulation replicates are presented in Tables 3, 4, 5, 6 and 7, and Appendix Tables S1-S2. For binary outcome with logit link, we do not report bias and empirical standard deviation because of the non-comparability of regression parameters from GLMM, GLMM2, and GEE. Note that no singular fit issue occurred in Scenario 1, so the GLMM2 with the two-step procedure was not relevant. For continuous outcomes with an identity link in Scenario 1, the three models produce equivalent point estimates, resulting in similar bias magnitude and empirical standard deviations across the models (Table 3). However, the GLMM leads to an inflated type I error rate of up to 21.8% (> 5%) due to the underestimation of the asymptotic standard error, resulting from covariance misspecification. In contrast, the GLMM2 produces the nominal type I error rate, while the GEE model shows an acceptable type I error rate, particularly when the number of clusters is 100. The empirical standard deviations of the regression coefficients estimates are similar in the GLMM2 and GLMM. However, the GEE model shows slightly less efficiency. A similar pattern is observed for count and binary outcomes, with GLMM having an inflated type I error rate of up to 47.2%.

**Table 3:** Simulation results of scenario 1 (adequate random effect and number of clusters): the inference for the null effect modification by outcomes and models

	Number of clusters = 50			Number of clusters = 100		
Models	Bias	ESD^a^	Type I error rate	Bias	ESD^a^	Type I error rate
Continuous						
GLMM2	0.010	0.072	0.054	0.003	0.051	0.046
GLMM	0.003	0.071	0.218	0.000	0.051	0.208
GEE	0.008	0.075	0.064	0.000	0.052	0.050
Count						
GLMM2	-0.015	0.237	0.069	0.001	0.168	0.059
GLMM	-0.010	0.264	0.472	0.001	0.187	0.465
GEE	-0.011	0.280	0.093	-0.003	0.190	0.062
Binary						
GLMM2	-	-	0.062	-	-	0.056
GLMM	-	-	0.187	-	-	0.181
GEE	-	-	0.069	-	-	0.057

^a^ESD=empirical standard deviation; GLMM2 is the true model in the data generation; Cluster size 100 is fixed; Wald t-test and z-test are used for GLMM/GLMM2 and GEE, respectively

In Scenario 2 with small random effects (Table 4), the GLMM2 is more likely to encounter singular fit issues than the GLMM (in up to 40% of simulation repetitions with count outcomes). The singularity issue is predominantly observed in the random slope estimates rather than the random intercept estimates. Increasing the number of clusters to 100 slightly reduces the rates of singularity across all three types of outcomes. The GLMM for binary outcome experiences a singular fit in a small number of simulations (75 and 28 when the number of clusters are 50 and 100, respectively) but not for continuous and count outcomes. Despite the singular fit, the point estimates of the three models are similar. However, when the number of clusters is 100, the GLMM2 and GEE conservatively control the type I error rate for the null effect modification compared to GLMM across all three outcomes. Most of the type I error rates of the two-step procedure meet the nominal level. When the random effects are small, the degree of type I error inflation in fitting the GLMM is reduced but still present.

**Table 4:** Simulation results of scenario 2 (small random effect but adequate number of clusters): the inference for the null effect modification by outcomes and models

	Number of clusters = 50			Number of clusters = 100		
Models	Bias	ESD^a^	Type I error rate	Bias	ESD^a^	Type I error rate
Continuous						
GLMM2	0.002	0.051	0.037 (248^b^)	-0.001	0.037	0.036 (183^b^)
Two-step	0.002	0.051	0.056	-0.001	0.037	0.044
GLMM	0.002	0.051	0.092 (0^c^)	-0.001	0.038	0.085 (0^c^)
GEE	0.002	0.052	0.053	-0.001	0.038	0.043
Count						
GLMM2	-0.002	0.127	0.055 (400^b^)	0.001	0.087	0.039 (286^b^)
Two-step	-0.002	0.127	0.067	0.001	0.087	0.050
GLMM	-0.002	0.127	0.085 (1^c^)	0.001	0.087	0.077 (0^c^)
GEE	-0.002	0.127	0.060	0.001	0.087	0.040
Binary						
GLMM2	-	-	0.056 (278^b^)	-	-	0.038 (218^b^)
Two-step	-	-	0.053	-	-	0.050
GLMM	-	-	0.071 (75^c^)	-	-	0.054 (28^c^)
GEE	-	-	0.076	-	-	0.045

^a^ESD=empirical standard deviation; ^b, c^= Number of singular fits out of 1,000 replicates; GLMM2 is the true model in the data generation; Cluster size 100 is fixed; Wald t-test and z-test are used for GLMM/GLMM2/Two-step and GEE, respectively

Scenario 3 reflects the data from the PROUD study (Table 5), specifically with 12 clusters of varying sizes. The small cluster correction methods described in Sect. Small Cluster Size. are applied. Compared to Scenario 2 across the three outcomes, the GLMM2s exhibit a decrease in the number of singular fits, and the GLMMs encounter no singular fit. Additionally, compared to GEE and GLMM, GLMM2 maintains the type I error rates close to the nominal level for the three outcome types and for both fixed and varying cluster sizes. In contrast, GEE has slightly inflated type I error rates (0.067–0.123) and the GLMM had very inflated type I error rates across all scenarios (0.138–0.449).

**Table 5:** Simulation results of scenario 3 (adequate random effect but small number of clusters): the inference for the null effect modification by outcomes and models

	Number of clusters = 12 with Fixed cluster size = 100			Number of clusters = 12 with varying cluster sizes of (25, 50, 100, 150, 300)		
Models	Bias	ESD^a^	Type I error rate	Bias	ESD^a^	Type I error rate
Continuous						
GLMM2^c^	-0.006	0.145	0.040 (159^b^)	0.007	0.154	0.048 (181^b^)
Two-step^c^	-0.006	0.145	0.053	0.007	0.157	0.089
GLMM^c^	-0.006	0.145	0.205	0.005	0.167	0.321
GEE^d^	-0.006	0.151	0.067	0.005	0.169	0.072
Count						
GLMM2^e^	-0.024	0.482	0.041 (17^b^)	0.001	0.507	0.034 (30^b^)
Two-step^e, f^	-0.024	0.483	0.045	-0.001	0.507	0.044
GLMM^f^	-0.018	0.523	0.356	0.013	0.584	0.449
GEE^d^	-0.019	0.525	0.099	0.011	0.586	0.123
Binary						
GLMM2^f^	-	-	0.060 (236^b^)	-	-	0.068 (167^b^)
Two-step^f^	-	-	0.090	-	-	0.144
GLMM^f^	-	-	0.138	-	-	0.210
GEE^d^	-	--	0.072	-	-	0.087

^a^ESD=Empirical standard deviation; ^b^=Number of singular fits out of 1,000 replicates; GLMM2 is the true model in the data generation; Wald t-test and F-test are used for GLMM/GLMM2/Two-step and GEE, respectively; ^c^ = Satterthwaite; ^d^ = Fary-Graubard; ^e^ = between-within; ^f^ = Subtracting the number of cluster-level parameters for fixed effects from the number of clusters

In Scenario 4 (Table 6), when the number of clusters is 50, with a fixed cluster size of 100, given that the GLMM is the true model in the data generation, GLMM effectively maintains the nominal type I error rate across the three outcomes. The GLMM2 and GEE also control the type I error rate at the nominal level for continuous outcomes. However, for count and binary outcomes, the GLMM2s slightly deflate the type I error rate (0.036–0.039), whereas the GEEs slightly inflate the type I error rates (0.063–0.069). In the PROUD-like setting, the GLMMs yield the nominal type I error rate for continuous and count outcomes but yield a slightly lower type I error rate for binary outcomes. The GLMM2s slightly deflate the type I error rate, whereas the GEE slightly inflates the type I error rates across the outcome types.

Scenario 5 includes two dummy variables representing a three-level categorical subgroup and evaluates only the GLMM and GLMM2, as the likelihood ratio test is used for the global test. In this scenario, GLMM2 estimates six parameters for the unstructured covariance matrix of the three subgroup-specific random effects, compared to just three parameters for the 2 × 2 covariance matrix in Scenarios 1–3. The simulation results are similar to those from Scenario 4, and the number of singular fits does not increase (Appendix Table S1). The main distinction is that both GLMM and GLMM2 (and consequently, the two-step procedure as well) exhibit more conservative control of type I error rates compared to Scenario 4 across all different settings. With the number of clusters 12, the likelihood ratio test without small-sample correction demonstrated similar control of the type I error rate compared to the Wald t-test with small-sample corrections in other scenarios. Thus, we did not apply small sample correction methods to the likelihood ratio test.

**Table 6:** Simulation results of scenario 4 (GLMM2 is over fitted): the inference for the null effect modification by outcomes and models

	Number of clusters (*N*) = 50 with fixed cluster size 100			Number of clusters (*N*) = 12 with varying cluster sizes of (25, 50, 100, 150, 300)		
Models	Bias	ESD^a^	Type I error rate	Bias	ESD^a^	Type I error rate
Continuous						
GLMM2	-0.001	0.046	0.045 (402^b^)	-0.002	0.090	0.032 (574^b^)
Two-step	-0.001	0.045	0.046	-0.002	0.089	0.038
GLMM	-0.001	0.046	0.058	-0.002	0.087	0.046
GEE	0.000	0.051	0.055	-0.001	0.097	0.062
Count						
GLMM2	0.001	0.099	0.036 (273^b^)	0.002	0.215	0.031 (387^b^)
Two-step	0.000	0.097	0.040	-0.003	0.212	0.042
GLMM	0.001	0.096	0.045	-0.002	0.203	0.051
GEE	0.002	0.104	0.063	0.002	0.214	0.077
Binary						
GLMM2	-	-	0.039 (173^b^)	-	-	0.041 (349^b^)
Two-step	-	-	0.039			0.043
GLMM	-	-	0.046	-	-	0.041
GEE	-	-	0.069	-	-	0.072

^a^ESD=empirical standard deviation; ^b^ = Number of singular fits out of 1,000 replicates The GLMM is the true model in the data generation; Wald t-test is used for GLMM/GLMM2/Two-step; for GEE, z-test and F-test are used for N=50 and N=12, respectively; when the number of clusters is 12, the small cluster correction methods were Satterwhite for continuous outcome across GLMM and GLMM2, parametric bootstrap for GLMM2 across count and binary outcomes, between-within for GLMM across count and binary outcomes and Fary-Graubard for GEE across the three outcomes

Scenario 6 (Table 7) assesses the robustness of GLMM2 under model misspecification, specifically when the heterogeneous covariances between arms for subgroup-specific random effects are ignored. As expected, the standard GLMM exhibits inflated type I error rates across all outcome types when the number of clusters is small or moderate. For continuous outcomes, both GLMM2 and the two-step procedure maintain the type I error rate at the nominal level. However, for count and binary outcomes, both approaches show slightly inflated type I error rates under the small and moderate clusters settings. Consistent with findings from other scenarios, GEE slightly inflates the type I error rate when the number of clusters is 12 and the Fay-Graubard small sample correction method is applied but controls it appropriately when the number of clusters is 50.

**Table 7:** Simulation results of scenario 6 (GLMM2 is misspecified): the inference for the null effect modification by outcomes and models

	Number of clusters (*N*) = 50 with fixed cluster size 100			Number of clusters (*N*) = 12 with varying cluster sizes of (25, 50, 100, 150, 300) ^c^		
Models	Bias	ESD^a^	Type I error rate	Bias	ESD^a^	Type I error rate
Continuous						
GLMM2	0.003	0.075	0.040 (1^b^)	0.007	0.164	0.048 (129^b^)
Two-step	0.003	0.075	0.040	0.006	0.165	0.080
GLMM	0.003	0.075	0.243	0.005	0.179	0.360
GEE	0.002	0.078	0.055	0.005	0.182	0.078
Count						
GLMM2	-0.006	0.248	0.070 (0^b^)	-0.006	0.541	0.040 (26^b^)
Two-step	-0.006	0.248	0.070	-0.004	0.541	0.043
GLMM	-0.006	0.283	0.518	0.014	0.639	0.484
GEE	-0.006	0.286	0.086	0.012	0.642	0.135
Binary						
GLMM2	-	-	0.074 (1^b^)	-	-	0.063 (123^b^)
Two-step	-	-	0.074	-	-	0.078
GLMM	-	-	0.219	-	-	0.210 (12^b^)
GEE	-	-	0.056	-	-	0.088

^a^ESD=empirical standard deviation; ^b^=Number of singular fits out of 1,000 replicates The GLMM2 with heterogenous covariances between arms for subgroup-specific random effects is the true model in the data generation; Wald t-test is used for GLMM/GLMM2/Two-step; for GEE, z-test and F-test are used for N=50 and N=12, respectively; ^c^=when the number of clusters is 12, the applied small cluster correction methods are Satterwhite for continuous and subtracting the number of cluster-level parameters for fixed effects from the number of clusters for binary outcome across GLMM and GLMM2; For count outcomes, subtracting the number of cluster-level parameters for fixed effects from the number of clusters and between-within degrees of freedom correction methods are applied for GLMM and GLMM2, respectively; Fary-Graubard correction is applied for GEE across the three outcomes

Scenario 7 compares the power of the standard GLMM, GLMM2, and the two-step procedure in settings where GLMM2 is overfitted. As expected, due to the trade-off between type I error rate control and statistical power, both GLMM2 and the two-step procedure demonstrate lower power compared to the standard GLMM when GLMM is the true model (Appendix Table S2).

### Results of real data application

The two clinics assigned to the intervention and usual care groups within each health system were nearly matched in size. However, the cluster sizes varied across health systems (median 121, range 12–434). On average, 52.7% of the patients in each clinic were women, with a range of 36.5% to 72.4%. The most notable difference among the three fitted models (Table 8) is that the intervention effect was found to be significantly different between men and women in the GLMM (p-value: 0.01), but not significantly different in the GLMM2 and GEE (p-values: 0.64 and 0.93, respectively). Nevertheless, the 95% confidence intervals for the PROUD intervention effects within men and women, as determined by GLMM, overlapped, consistent with the findings of the GLMM2 and GEE. This difference may be attributed to potential heterogeneous within-cluster correlations by sex, which may have led to underestimation of standard errors. In each simulation of Scenario 3, the effect modification estimate from the GLMM was closer to the estimate from GEE, compared to the GLMM2’s estimate; yet, all the effect modification estimates are asymptotically unbiased for their respective target estimand. Conversely, in this application we see that the effect modification estimates from the three models are slightly different. Although small, these discrepancies in the effect modification estimates among the three fitted models could be attributed to sex imbalances across clinics, informative clinic sizes or that the different approaches are estimating slightly different quantities since the GLMM methods do not weight participants equally (patients in smaller clinics have more weight than larger clinics) relative to GEE with independent working correlation does weight equally.

**Table 8:** Comparison of three models applied to the PROUD data

	GLMM2			GLMM			GEE		
Fixed effects	Beta	Standard Error	t-test P-value	Beta	Standard Error	t-test P-value	Beta	Standard Error	F-test P-value
Intercept	-5.34	0.30	< 0.001^a^	-5.38	0.26	< 0.001^b^	-4.94	0.09	< 0.001^c^
Intervention PROUD	-0.067	0.43	0.88^a^	0.21	0.37	0.58^b^	-0.20	0.25	0.19^c^
Male	-0.090	0.32	0.79^a^	0.015	0.03	0.60^b^	-0.04	0.06	0.83^c^
Baseline Outcome	0.28	0.003	< 0.001^a^	0.27	0.003	< 0.001^b^	0.28	0.02	< 0.001^c^
Effect modification (Inter × Male)	0.21	0.45	0.64^a^	-0.14	0.04	0.01^b^	0.02	0.14	0.93^c^

Intervention effect by subgroup	Effect (95% Confidence interval^a^)	Effect (95% Confidence interval^b^)	Effect (95% Confidence interval^c^)
Within men	0.14 (-0.84, 1.13)	0.072 (-0.75, 0.89)	-0.18 (-0.60, 0.24)
Within women	-0.067 (-1.02, 0.89)	0.21 (-0.61, 1.03)	-0.20 (-0.51. 0.12)

^a^=Between-within correction; ^b^= Subtracting the number of cluster-level parameters for fixed effects from the number of clusters; ^c^= Fay-Graubard

## Discussion

This article spotlights a major analytical challenge that can arise when inferring effect modification by subgroups using clustered data. Focusing on the CRT setting, we found that ignoring subgroup-specific correlations within clusters can lead to severe type I error inflation in GLMM when testing for effect modification (up to 47.2% across our simulation scenarios versus the nominal 5% level). To address this issue, we proposed a GLMM2 that accounts for subgroup-specific correlations in addition to within-cluster correlations. In the “ideal” CRT setting (e.g., having ≥ 50 clusters with non-trivial random effects) the proposed GLMM2 controlled the type I error rate at the nominal level as did GEE (though rates were slightly elevated even with as many as 50 clusters). When there are truly no subgroup-specific correlations within clusters (i.e., the standard GLMM is the true model and the GLMM2 is over fitted), the type I error rate under the GLMM2 was conservatively controlled. When the true model is unknown in practice, GLMM2 leads to loss of statistical power when it is over fitted. However, appropriately controlling of the type I error rate should be prioritized in clinical trials.

We also examined the impact of misspecified GLMMs under more challenging scenarios of CRTs, including scenarios with few clusters or small random effects. With small random effects, the GLMM2 conservatively controls the type I error rate and may result in a singular fit. We therefore recommend considering a two-step procedure that subsequently fits the standard GLMM model after encountering a singular fit when fitting the GLMM2. In scenarios with a small number of clusters, the GLMM2 utilizing small-sample correction methods for Wald t-test yielded type I errors closer to the nominal level compared to the standard GLMM. However, it still exhibited some inflation for binary outcomes, albeit smaller than the Type I error inflation observed with GEE.

When the data-generating model in the simulation follows the GLMM2 structure, the GEE with an independent working correlation is technically misspecified, even though it employs a robust sandwich variance estimator. Given our focus on type I error control rather than power, and the fact that GEE is a widely used, robust approach requiring only correct specification of the first and second moments, we included it in comparisons alongside the conditional models, GLMM and GLMM2. Overall, GEE maintains type I error rates close to the nominal level across all scenarios when the number of clusters exceeds 50. However, GEE with small sample correction tends to exhibit slight inflation in type I error rates when the number of clusters is 50 or fewer.

The robustness of GLMM2 was evaluated in two settings: when the model is misspecified, and when it includes dummy variables for a three-level categorical subgroup, which increases the number of covariance parameters to estimate. When GLMM2 is misspecified, it maintains good control of the type I error rate for continuous outcomes, while showing slight inflation (6–7%) for count and binary outcomes. However, this inflation is substantially smaller than that observed under a misspecified standard GLMM in other scenarios. The type I error control of GLMM2 with two dummy variables for a three-level categorical subgroup is comparable to its performance with a binary subgroup. However, as the number of subgroup levels increases, the additional number of parameters to estimate in the unstructured covariance matrix also grows—specifically, by 𝑝+1, where 𝑝 is the total number of subgroup levels. Therefore, it is important to carefully consider the appropriate number of levels for categorical subgroups using dummy variables when applying GLMM2.

Based on our simulation study using Wald t-test, we identified which correction method for studies with few clusters performed best for flexible and standard GLMMs when the true model is known. These correction methods demonstrated acceptable control of type I errors close to the nominal level. In practical applications, where the true correlation structure within clusters is unknown, we recommend applying the selected correction methods for both the GLMM2 and the standard GLMM. If the results obtained using these methods differ significantly, we advise reporting results of GLMM2. The various small-sample correction methods recommended for each combination of fitted and true models include quite complex and ad-hoc approaches. Such complexity underscores the necessity for future research to evaluate and propose robust methods applicable to both binary and count outcomes, irrespective of the true model. This could involve considering wild bootstrap resampling methods to obtain the empirical distribution of null effect modification [21]. In our simulation scenario considering a subgroup with more than 2 levels, the likelihood ratio test for the global null hypothesis generally maintained appropriate type I error control. However, most small sample correction methods are designed for the Wald t-test. Thus, further research is warranted to evaluate potential small-sample correction strategies tailored to the likelihood ratio test based on Chi-square distributions.

## Conclusion

In conclusion, we recommend that studies evaluating HTE in settings with clustered data carefully consider the correlation structure when using GLMM methods. For CRTs, we recommend using GEE or GLMM2 that allows for separate within-cluster correlations across subgroups. Future work is needed to examine the performance of the methods in other settings of clustered data (e.g., multiple layers of clustering, and settings with imbalanced subgroups across clusters).

## Supplementary Information

Supplementary Material 1.

## Data Availability

No datasets were generated or analysed during the current study.

## Abbreviations

CRT: Cluster randomized trial
CTN: Clinical Trials Network
GEE: Generalized estimating equation
GLMM: Generalized linear mixed-effects model
HTE: Heterogeneity of treatment effect
LMM: Linear mixed-effects model
LRT: Likelihood ratio test
NIDA: National Institute for Drug Abuse
PROUD: Primary Care Opioid Use Disorder treatment
RCT: Randomized clinical trials
