# Supplementary material for: Increased risk of type I errors for detecting heterogeneity of treatment effects in cluster-randomized trials using mixed-effect models
Source: BMC Med Res Methodol. 2026 Jan 12;26:30. doi: 10.1186/s12874-025-02744-6 (PMC12888205; doi:10.1186/s12874-025-02744-6)
Supplement: Supplementary file 1 — Supplementary Material 1. [file 12874_2025_2744_MOESM1_ESM.docx]

## Appendix

We provide example R code implementing GEE, GLMM and the GLMM2 for a single generated dataset with a count outcome.

library(mvtnorm)

library(mvProbit)

library(lme4)

library(gee)

## Function of generating count data

count.gen<-function(nc,cs,beta,V){

out<-NULL

trtlist=c(rep(0,nc/2),rep(1,nc/2))

trt.assign<-sample(1:nc,replace=FALSE)

for(i in 1:nc){

sid<-seq(1,cs)+(i-1)*cs

trt=trtlist[trt.assign[i]]

sex<-rbinom(n=cs,size=1,prob=c(0.5))

ns1=sum((sex==1)+0)

ns0=sum((sex==0)+0)

u<-rmvnorm(n=1,mean=rep(0,2),sigma=V)

re<-rep(NA,cs)

re[sex==1]<-u[1]

re[sex==0]<-u[2]

model=exp(beta[1]+beta[2]*trt+beta[3]*sex+re)

y=rpois(cs,model)

out<-rbind(out,data.frame(clinic_id=i,sid,y,trt=trt,sex))

}

return(out)

}

#parameters setting

#number of clusters

nc=50;

#cluster size/

cs=100;

V=symMatrix( c( 0.5,0.25,0.5 ) )

beta<-c(-1, -0.07, -0.5)

# Generating count data

sdata<-count.gen(nc,cs,beta=beta,V)

sdata$sex<-factor(sdata$sex)

# Fit GLMM

fit<-glmer(y ~ trt+sex+trt*sex+ (1|clinic_id), data=sdata, family=poisson,

control = glmerControl(optCtrl = list(maxfun = 10000)))

# Fit GLMM2: in this code, we uses random effects for the intercept and slope of sex. This re-parametrization is equivalent to modeling subgroup-specific random intercept effects when sex are dummy variables.

fit2<-glmer(y ~ 1+trt+sex+trt*sex+ (1+sex|clinic_id), data=sdata, family=poisson,

control = glmerControl(optCtrl = list(maxfun = 10000)))

# Fit GEE

fit3<-gee(y ~ 1+trt+sex+trt*sex,id=clinic_id,data=sdata,family="poisson")

## Simulation Results

Table S1. Simulation results of Scenario 5 using likelihood ratio test with a categorical subgroup of three levels (GLMM2 is over fitted): the inference for the global test regarding the null effect modifications by outcomes and models.

|  | Number of clusters=50 with fixed cluster size 100 | | Number of clusters=12 with varying cluster sizes of (25, 50, 100, 150, 300) | | |
| --- | --- | --- | --- | --- | --- |
| Models | Type I error rate | | Type I error rate | |  |
| **Continuous** |  |  | |  |  |
| GLMM2 | 0.041 (612^b^) |  | | 0.037 (778^b^) |  |
| Two-step | 0.045 |  | | 0.051 |  |
| GLMM | 0.052 |  | | 0.055 |  |
| **Count** |  |  | |  |  |
| GLMM2 | 0.044 (118^b^) |  | | 0.033 (13^b^) |  |
| Two-step | 0.045 |  | | 0.034 |  |
| GLMM | 0.050 |  | | 0.048 (1 ^b^) |  |
| **Binary** |  |  | |  |  |
| GLMM2 | 0.037 (35^b^) |  | | 0.027 (116^b^) |  |
| Two-step | 0.037 |  | | 0.032 |  |
| GLMM | 0.041 |  | | 0.053 |  |

^a^ESD=empirical standard deviation; ^b^=Number of singular fits out of 1,000 replicates. The GLMM is the true model in the data generation. Likelihood ratio test for the global test was applied to all outcomes.

Table S2. Simulation results of Scenario 7 using Wald t-test (GLMM2 is over fitted): the power for the non-null effect modification by outcomes and models.

|  | Number of clusters=50 with fixed cluster size 100 | | | ­ | Number of clusters=12 with varying cluster sizes of (25, 50, 100, 150, 300) | | |
| --- | --- | --- | --- | --- | --- | --- | --- |
| Models | Bias | ESD^a^ | Power |  | Bias | ESD^a^ | Power |
| **Continuous** |  |  |  |  |  |  |  |
| GLMM2 | -0.001 | 0.046 | 0.989 (414^b^) |  | -0.002 | 0.090 | 0.469 (576^b^) |
| Two-step | -0.001 | 0.046 | 0.989 |  | -0.002 | 0.089 | 0.501 |
| GLMM | -0.001 | 0.046 | 0.992 |  | -0.002 | 0.087 | 0.627 |
| **Count** |  |  |  |  |  |  |  |
| GLMM2 | 0.002 | 0.096 | 0.162 (290^b^) |  | 0.006 | 0.212 | 0.061 (358^b^) |
| Two-step | 0.002 | 0.096 | 0.163 |  | 0.006 | 0.206 | 0.070 |
| GLMM | 0.001 | 0.095 | 0.184 |  | 0.002 | 0.200 | 0.088 |
| **Binary** |  |  |  |  |  |  |  |
| GLMM2 | 0.011 | 0.160 | 0.115 (444^b^) |  | -0.004 | 0.334 | 0.057 (297^b^) |
| Two-step | 0.012 | 0.160 | 0.145 |  | -0.005 | 0.330 | 0.062 |
| GLMM | 0.012 | 0.160 | 0.149 (8 ^b^) |  | -0.002 | 0.145 | 0.162 |

^a^ESD=empirical standard deviation; ^b^=Number of singular fits out of 1,000 replicates The GLMM is the true model in the data generation; when the number of clusters is 12, the small cluster correction methods were Satterwhite for continuous outcome across GLMM and GLMM2, parametric bootstrap for GLMM2 across count and binary outcomes, between-within for GLMM across count and binary outcomes.
